# Selected MicroRNAs Define Cell Fate Determination of Murine Central Memory CD8 T Cells

**DOI:** 10.1371/journal.pone.0011243

**Published:** 2010-06-22

**Authors:** Gonzalo Almanza, Antonio Fernandez, Stefano Volinia, Xochitl Cortez-Gonzalez, Carlo M. Croce, Maurizio Zanetti

**Affiliations:** 1 The Laboratory of Immunology, Department of Medicine and Moores Cancer Center, University of California San Diego, La Jolla, California, United States of America; 2 Department of Molecular Virology, Immunology, and Medical Genetics and Comprehensive Cancer Center, Ohio State University, Columbus, Ohio, United States of America; 3 Telethon Facility-Data Mining for Analysis of DNA Microarrays, Department of Morphology and Embryology, University of Ferrara, Ferrara, Italy; New York University, United States of America

## Abstract

During an immune response T cells enter memory fate determination, a program that divides them into two main populations: effector memory and central memory T cells. Since in many systems protection appears to be preferentially mediated by T cells of the central memory it is important to understand when and how fate determination takes place. To date, cell intrinsic molecular events that determine their differentiation remains unclear. MicroRNAs are a class of small, evolutionarily conserved RNA molecules that negatively regulate gene expression, causing translational repression and/or messenger RNA degradation. Here, using an *in vitro* system where activated CD8 T cells driven by IL-2 or IL-15 become either effector memory or central memory cells, we assessed the role of microRNAs in memory T cell fate determination. We found that fate determination to central memory T cells is under the balancing effects of a discrete number of microRNAs including miR-150, miR-155 and the let-7 family. Based on miR-150 a new target, KChIP.1 (K ^+^ channel interacting protein 1), was uncovered, which is specifically upregulated in developing central memory CD8 T cells. Our studies indicate that cell fate determination such as surface phenotype and self-renewal may be decided at the pre-effector stage on the basis of the balancing effects of a discrete number of microRNAs. These results may have implications for the development of T cell vaccines and T cell-based adoptive therapies.

## Introduction

The defense against pathogens and cancer requires T cell immunity. T cells see antigen presented in association to molecules of the Major Histocompatibility Complex (MHC) on specialized cells, dendritic cells, macrophages and B cells. Their activation requires an additional signal from costimulatory molecules. Once T cell recognition initiates, the primary T cell response follows temporal characteristics that are well understood. Presumably through symmetric cell division T cells expand clonally for about 7 days in a way that is proportional to the antigen dose [1], and then contract through a program that is independent of the magnitude of expansion [2]. During the contraction phase the majority (90–95%) of effector T cells die by apoptosis. One view is that memory T cells are generated at this time, even though the events are not entirely understood. An alternative view is that memory T cells originate directly from naïve T cells that undergo asymmetric cell division after prolonged contact with the antigen presenting cell [3]. It is assumed that in both instances memory T cells perpetuate thereafter either through self-renewal, a stem-cell like property, or by homeostatic proliferation.

From the outset, memory T cells enter a fate determination program that divides them into two main populations on the basis of surface phenotype: effector memory (CD44^+^/CD62L^low-nil^/CCR7^nil^) and central memory (CD44^+^/CD62L^hi^/CCR7^hi^) T cells. These two lineages have distinct homing characteristics and functional properties. Studies in humans suggest that effector memory and central memory T cells form two independent populations [4]. In contrast, studies in the mouse suggest that they may be part of a linear developmental program where effector memory cells can convert into central memory cells [5]. Since in many systems protection appears to be preferentially mediated by T cells of the central memory type [6], [7], [8], [9], it is important to understand when and how lineage differentiation begins (the lineage differentiation problem). Resolving this issue has direct implications for vaccine design.

In past years studies to resolve the lineage differentiation problem have underscored the importance of antigen dose, the degree of inflammation at the time of priming, and the frequency of naïve precursors [10]. Efforts to deconvolute in molecular terms fate determination in memory T cells have been pursued independently through micro-array gene profiling [11], [12]. Studies in the mouse have concluded that antigen specific CD8 T cells acquire memory properties several weeks after antigen clearance, suggesting that memory T cells arise from effector cells. This is in line with T cell marking experiments that found that memory T cells derive from effector T cells [13], [14], [15]. However, gene profiling has not been able to identify a precise time or a set of transcriptional events that more directly associate with fate determination of effector memory and central memory T cells.

**Figure 1:**
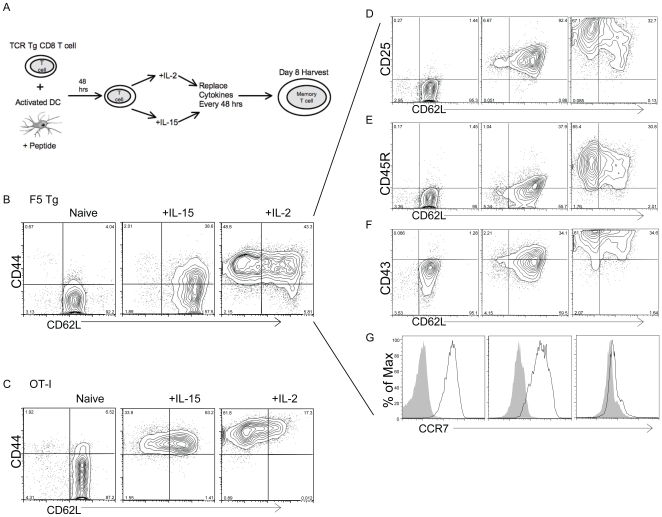
Model and phenotypic analysis of *in vitro* generated F5 and OT-I memory CD8 T cells. (A) Experimental model to study fate determination of memory CD8 T cells. Spleens from naïve F5 or OT-I transgenic mice were harvested and cultured with LPS blasts, activated dendritic cells (DC), pulsed with peptide (NP_366–374_ for F5 or Ova_257–264_ for OT-I T cells). After 48 hrs, LPS blasts were removed and activated cells were re-cultured with either IL-15 (40 ng/mL) or IL-2 (40 ng/mL). Every 48 hrs the media was replenished with fresh cytokines at 20 ng/mL. (B–C) Expression of CD44 (IM7) and CD62L (MEL-14) on naive and day-8 IL-15- or IL-2-derived F5 or OT-I CD8 T cells using anti-mouse CD44-PE and rat anti-mouse CD62L-APC. Cells were stained with 7-ADD (7-AAD PerCP) to identify dead cells, then gated on CD8 T cells. Each histogram represents the analysis of 10,000 7-ADD-negative CD8 T cells. (D–F) Expression of CD25 (PC61), CD45R (B220), CD43 (IB11) and CD62L (MEL-14) on naïve and day-8 IL-15- or IL-2-derived F5 Tg CD8+ T cells. Numbers represent percentage of cells in the respective quadrants. (G) Expression of CCR7 (4B12) on naïve and day-8 IL-15- or IL-2-derived F5 CD8 T cells. Filled histograms  =  isotype (rat IgG2a) staining; solid black line = CCR7 staining.

MicroRNAs are a class of small, evolutionarily conserved, RNA molecules that negatively regulate gene expression, causing translational repression and/or messenger RNA degradation [16], [17]. MicroRNAs have been implicated in the control of many fundamental cellular and physiological processes, directly or indirectly [18]. Studies comparing naïve, effector and memory CD8 T cells, show that a small set of microRNAs is downregulated in effector T cells compared to naive cells, but also that expression tends to come back in memory T cells [19]. However, direct comparison between the effector memory and central memory subsets has not been performed. Here we used microRNA analysis to verify if regulation at this level is involved in memory T cell fate determination. To better control the temporal dynamics of memory generation, we used an *in vitro* system of effector memory/central memory CD8 T cell differentiation where naïve T cells move from antigen activation into fate determination on the basis of cytokine selection in culture (Figure 1, A). CD8 T cells exiting *in vitro* education with IL-2 or IL-15 acquire the migratory and functional properties characteristic of effector memory and central memory CD8 T cells generated *in vivo* [20], [21]. Here, we profiled microRNAs of IL-2 and IL-15 derived memory CD8 T cells in an attempt to identify variations in expression associated with the tempo of cell fate determination.

## Results

### Memory Lineage Differentiation in TCR transgenic T cells of different specificity

We used an *in vitro* system to establish the generation of effector memory and central memory CD8 T cells starting from naive TCR transgenic CD8 T cells [20]. To minimize potential bias we studied in parallel two different TCR transgenic T cells: CD8 T cells from RAG −/− F5 transgenic mice (F5) that are specific for the ASNENMDAM peptide of the nucleoprotein (NP) antigen of the influenza A virus [22], and CD8 T cells from OT-I mice specific for the SIINFEKL peptide of ovalbumin [23]. We reasoned that microRNA expression levels that are truly associated with the process of cell fate determination of memory T cells would be shared by T cells with distinct TCR specificities.

Activation of naïve F5 and OTI CD8 T cells followed by *in vitro* culture either in IL-2 or IL-15 (Fig. 1, A) yields memory T cells with effector memory (CD44^hi^/CD62L^lo/nil^) or central memory (CD44^hi^/CD62L^hi^) characteristics by day 8, that is six days after cytokine-directed memory lineage differentiation. Detailed surface phenotype analysis of day 8 memory T cells showed that IL-15-derived memory T cells are in addition CD25 intermediate, CD45R (B220)^lo^, CD43^lo^ and CCR7^+^. In contrast IL-2-derived memory T cells are CD25^hi^, CD45^int/hi^, CD43^hi^ and CCR7^−^. A phenotypic analysis of OT-I cells showed comparable results on day 8. Interestingly, a comparison of the tempo of central memory T cell generation in F5 and OT-I cells showed consistently that whereas the percentage of CD44^hi^/CD62L^hi^ OT-I cells increases progressively through day 8, the percentage of CD44^hi^/CD62^hi^ F5 cells is maximal at day 4 and decreases thereafter (Figure S1). This suggests different modalities in the generation of the central memory phenotype, possibly reflecting different avidity of either the TCR or the peptides for the MHC.

### MicroRNA Analysis of IL-2 and IL-15 directed CD8 memory T cell fate determination

**Figure 2:**
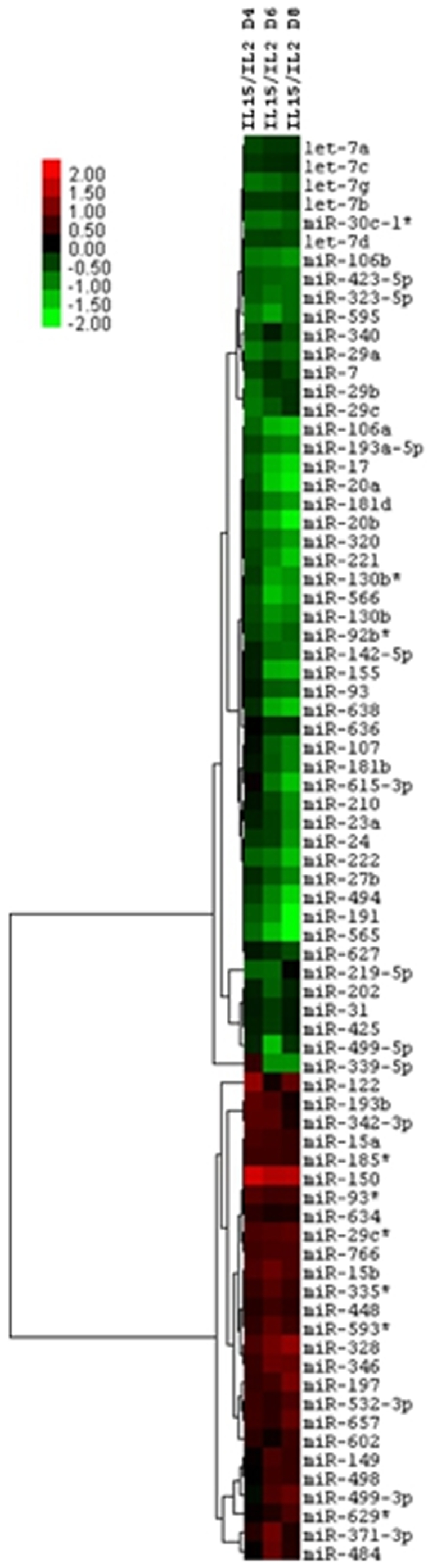
MicroRNA expression analysis in fate determination of central and effector memory CD8 T cells. Clustering tree representation of differences in microRNA expression between effector memory and central memory CD8 T cells. Total RNA was extracted from cells at different time points for either IL-15 or IL-2 treatments. Samples were run in triplicate. Six independent experiments were performed, three with F5 and three with OTI CD8 T cells. Cluster and TreeView were used to plot the log_2_ IL-15/IL-2 expression ratios. Red square means higher miR-NA expression upon IL-15 (IL-15/IL-2>1) and green squares mean higher miR-NA expression upon IL-2 treatment (IL-2/IL-15>1). The values are log_2_ ratios, i.e. 0 (black) means the fold change (or ratio) between IL-15 and IL-2 signals is 1 (no difference).

We identified the microRNAs differentially expressed upon IL-2 and IL-15 treatment by using paired t-test for each experimental time point. Prior to t-test, microRNA datasets were quantile-normalized and the invariant microRNAs removed. A microRNA was considered statistically significant if its t-test p value was <0.05. False detection rates helped to control for multiple testing. Six independent experiments were performed, three with F5 T cells and three with OT-I T cells, and total RNAs were collected on day 0, 2, 4, 6 and 8. We measured the changes in microRNA profile after treatment with IL-2 or IL-15 and found different microRNA profiles for IL-15 (Figure S2,A) and IL-2 (Figure S2,B), respectively. We then combined the effect of the two treatments into a single clustering tree (Fig. 2) representing the log_2_ ratios of mean IL15/IL2 expression at each time point for the significant microRNAs. The expression at days 4, 6 and 8 was measured relative to the baseline values (day 0 and 2). We noted that differentiation in the presence of IL-15 induced a differential increase of miR-150 (red squares) and a decrease of miR-155. In converse, differentiation in the presence of IL-2 down-modulated (green squares) miR-150 and miR-146, albeit the effect on miR-146 was late. Of note, we found that members of the let-7 family (let-7 a, b, c, d and g) were all consistently down-regulated (∼30%) in IL-15 treated T cells (Fig. 2 and Figure S2,A).

### Expression of miR-150 and miR-155 in central memory T cells by qPCR

**Figure 3:**
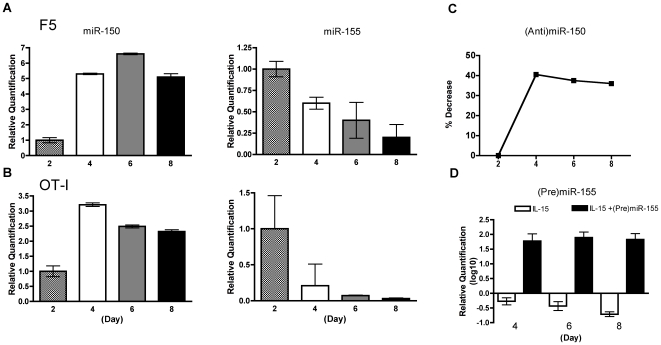
Levels of miR-150 and miR-155 expression in central memory CD8 T cells by qPCR. Fold modulation in F5 (**A**) and OT-I (**B**) IL-15-derived memory CD8 T cells. Total RNA was extracted from triplicate cultures on day 0, 2, 4, 6 and 8, and subjected to microRNA-specific reverse transcription (RT) followed by qPCR for miR-150 and miR-155 with snoRNA202 as endogenous control. Data points refer to the mean ± SD of a combined experiment with F5 and OT-I T cells and are representative of two independent experiments for F5 and OT-I T cells, respectively. (C) Decrease in miR-150 expression by the inhibitory microRNA precursor (anti)miR-150 (ABI AM150). Briefly, day-2 peptide-activated CD8 T cells were transfected with AM150 (150 nM) and cultured as indicated in Materials and Methods. On day 4, 6 and 8, cells were harvested, total RNA extracted, and levels of miR-150 expression measured by qPCR and compared with day-2 baseline values. Representative of two independent experiments. Tests were done in triplicate. (D) Reversal of miR-155 expression levels in central memory T cells by the stimulatory microRNA precursor (pre)miR-155 (ABI PM155). Day-2 peptide-activated CD8 T cells were transfected with PM155 (150 nM). Cells were then cultured, harvested, and the RNA tested as indicated above. Data point refers to the combined mean ± SD of two independent experiments with F5 and OT-I T cells, respectively. Tests were done in triplicate.

The miRNA array data reflected the average of six independent experiments and included, as noted above, two different types of TCR transgenic T cells. The expression of miR-150 and miR-155 in the IL-15-directed differentiation of central memory T cells was verified by qPCR using day-2 values as the baseline reference. We reasoned that any meaningful variation would exclude the broad variations in microRNA expression due to T cell activation. miR-150 was elevated in both F5 and OT-I IL-15-derived memory T cells. The increased expression was immediate and persisted through day 8 (Fig. 3,A). The increase in expression was similar in F5 and OT-I T cells. Similarly, the decreased expression of miR-155 was confirmed both in F5 and OT-I cells (Fig. 3,B). The decrease was progressive and maximal at day 8, consistent with the array data. To see if these variations in miRNA expression were specific and, more importantly, could be modulated, experiments were repeated where activated T cells were transfected with inhibitory (anti-)miR-150 or stimulatory (pre-)miR-155 precursors on day 2 before culture with IL-15. Transfection with (anti-)miR-150 inhibited the increased expression of miR-150 by 40% (Fig. 3,C). On the other hand, transfection with (pre-)miR-155 caused a dramatic upregulation of miR-155 expression at all time points (Fig. 3,D). Collectively, the data suggest that during the generation of central memory CD8 T cells two microRNAs, miR-150 and miR-155, may play a reciprocal non-static role in the progression of CD8 T cells to central memory phenotype.

### Target prediction uncovers a new gene in the differentiation of central memory CD8 T cells

**Figure 4:**
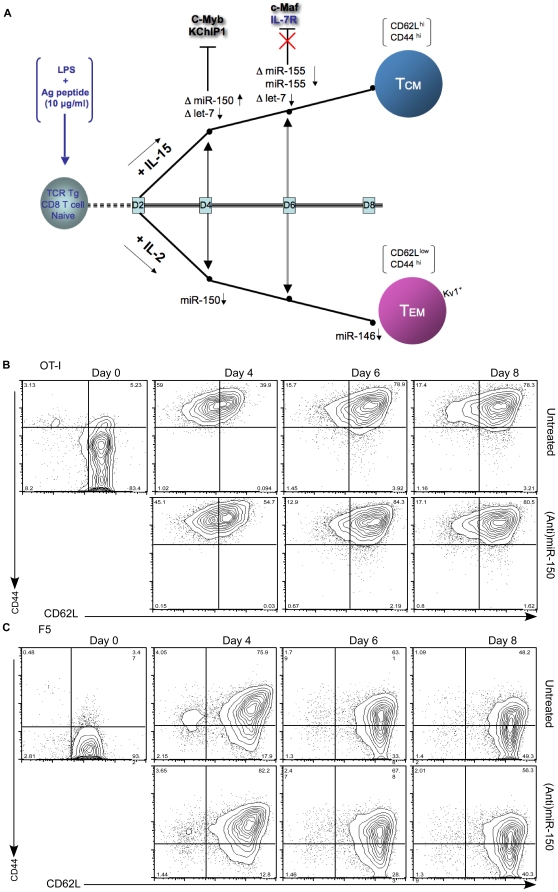
Effect of microRNAs in fate determination of memory CD8 T cells. (A) Model and findings. Memory T cell fate determination is graphically represented as two trajectories originating from a common start point: the day-2 peptide-activated CD8 T cells. Identified on the trajectories are the observed variations in miR-150 and miR-155 expression, and the target genes considered. ↑and↓ refer to net increase/decrease from the corresponding baseline values. Δ refers to a variation between IL-15-derived and IL-2-derived memory T cells at corresponding time points. (B–C) Kinetics of phenotypic changes in central memory development originating from antigen-activated OT-I and F5 CD8 T cells with or without (anti)miR-150. Spleens from naïve OT-I and F5 cells transgenic mice were harvested and cultured with DC pulsed with peptide (Ova_257–264_ for OT-I or NP_366–374_ for F5). After 48 hrs, DC were removed and activated T cells were re-cultured with IL-15 (40 ng/mL). In parallel cultures CD8 T cells were transfected with 150 nM of (anti)miR-150 as indicated in Materials and Methods. Every 48 hrs the media was replenished with fresh IL-15 (20 ng/mL). Expression of CD44 (IM7) and CD62L (MEL-14) was detected using anti-mouse CD44-PE and rat anti-mouse CD62L-APC, respectively. Cells were stained with 7-ADD (7-ADD PerCP) to identify dead cells, then gated on CD8 T cells. Each histogram represents the analysis of 10,000 7-ADD-negative CD8 T cells. Numbers represent percentage of cells in the respective quadrants.

**Figure 5:**
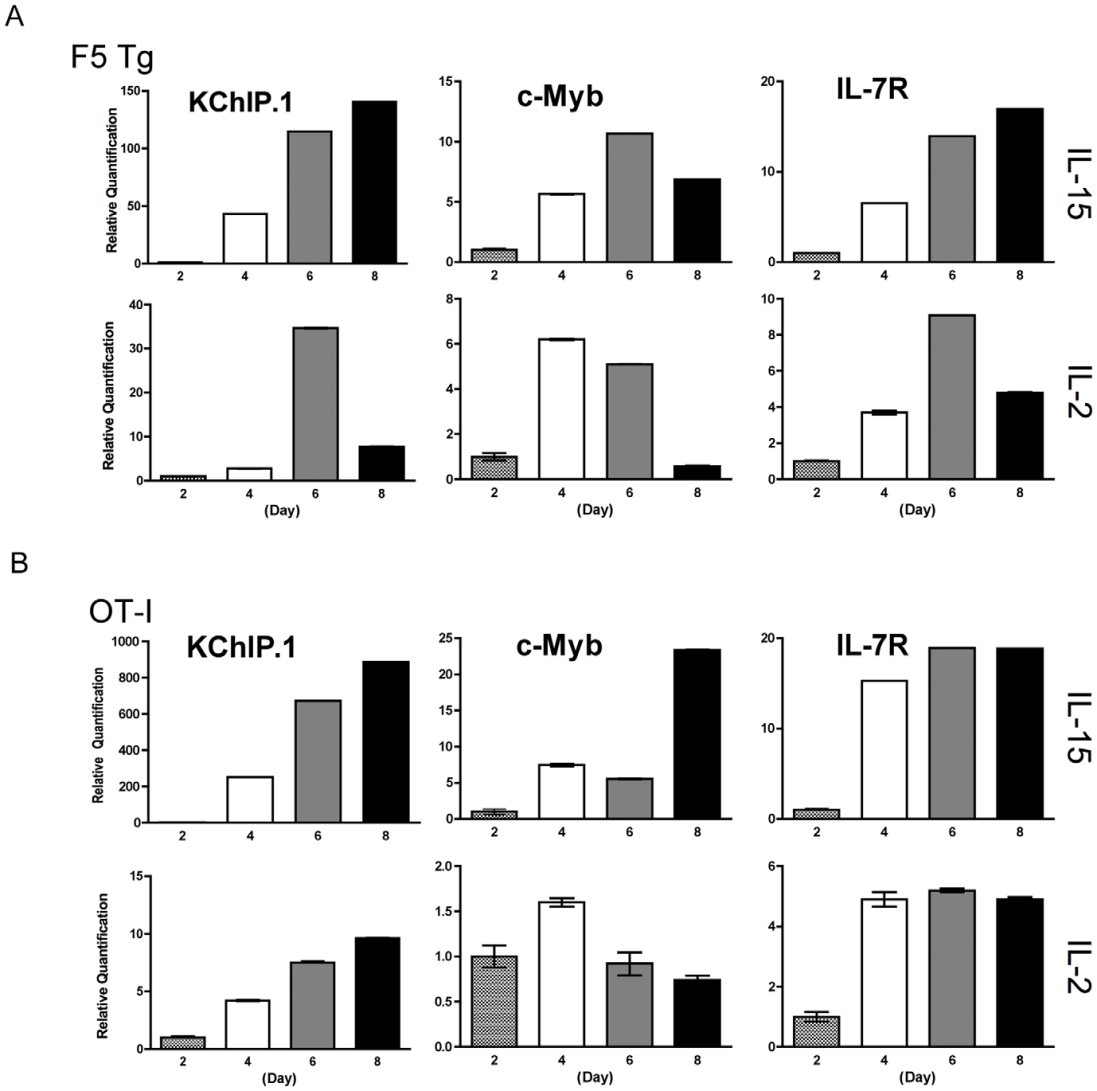
Expression of microRNA-predicted target genes by qPCR. Expression of KChIP.1, c-Myb and IL-7R was quantitated in total RNA extracted from F5 (A) or OT-I (B) CD8 T cells cultured in IL-15 or IL-2. Reverse transcription was performed starting with the same amount of RNA template to generate cDNA. KChIP.1 and IL-7R were amplified using custom-made probes and primer sets. c-Myb was amplified with ABI probes Mm01188144_m1. Data are representative of two independent experiments with F5 and OT-I T cells each. Tests were done in triplicate.

MicroRNAs exert transcriptional/post transcriptional gene expression regulation through binding to partially complementary sites in the 3″-untranslated region of target genes. We used PicTar [24], a target prediction algorithm, to identify the potential targets for miR-150 and miR-155 with a prima facie relation to T lymphocytes. These predictions are plotted along the trajectories of IL-15 and IL-2-directed memory T cell generation (Fig. 4). miR-150 predicts two targets, KChIP.1 (K ^+^ channel interacting protein 1) and c-Myb. KChIP.1, a member of a family of Ca^++^ -binding protein that binds to the intracellular N-terminal domain of A-type potassium channels [25], and is expressed in T lymphocytes where it represses IL-2, IL-4 and IFNγ production during antigen specific activation [26]. KChIP.1 was readily and highly up-regulated in both F5 and OT-I IL-15-directed memory T cells (maximal amplification = 150-fold in F5 T cells and >1000-fold in OT-I cells when compared to the respective day-2 baseline value). KChIP.1 up-regulation appeared to be specific for central memory T cell differentiation since no up-regulation occurred when memory T cells were generated with low dose IL-2, except a small isolated increase on day 6 (Fig. 5,A and B), whose significance remains unclear. c-Myb is a key transcription factor in lymphocyte development. In the development of B cells a miR-150/c-Myb partnership has already been demonstrated [27], [28]. Furthermore, c-Myb has been shown to play a role in the development of thymocytes but also in the proliferative response of mature T cells [29]. Here, c-Myb was up-regulated in both F5 Tg and OT-I IL-15-derived memory T cells. While there was no c-Myb up-regulation in IL-2-derived OT-I cells, F5 T cells showed up-regulation on average 2-fold lower than their IL-15 counterpart (Fig. 5,A and B). Previously it was shown that purified splenic CD8T cells from c-Myb knock-out mice have a 3-fold greater response to anti-CD3/anti-CD28 or IL-2 [29], implying regulation of mature CD8 T cells by cMyb. Whereas the role of IL-15 was not investigated, our results provisionally suggest that c-Myb plays a differential role in proliferation and fate determination of memory CD8 T cells depending on whether they respond to IL-15 or IL-2. Further studies are needed to elucidate this issue.

Our model (Fig. 4,A) also predicts IL-7R to be a target of miR-155. IL-7R is expressed on both central memory (CD62L^hi^) and effector memory (CD62L^lo^) CD8 T cells. IL-7R plays a central role in positive selection in the thymus [30], and enhances the survival of mature T cells including memory T cells [31]. However, whether it identifies memory T cell precursors is still matter of debate. Relative quantification shows a greater fold increase in central memory than in effector memory T cells (Fig. 5,A and B), suggesting that IL-15 signaling is intrinsically more effective in dictating IL-7R expression. This is consistent with the observed decreased level of miR-155 in central memory T cells.

### Anti-miR-150 modulates the central memory phenotype

We decided to verify whether the acquisition of a central memory phenotype could be modulated by treating antigen-activated, day-2 CD8 T cells with the (anti)miR-150. F5 or OT-I T cells were transfected with (anti)miR-150, cultured in the presence of IL-15 and harvested every 2 days. A marked effect was noted in OT-I cells on day 4 where down-modulation of miR-150 was followed by an increase in the percentage of central memory T cells ∼30% (39−>54%) (Fig. 4,B). The positive effect on the percentage of cells with central memory phenotype was maintained through day 8, albeit at a smaller magnitude. The effect on F5 T cells was overall less pronounced ∼10% increase (75−>82%), which is explained by the different kinetics between F5 and OT-I cells (Figure S1). Combined with the prediction that miR-150 negatively regulates KChiP.1, these results suggest that miR150 exerts negative feedback regulation on the acquisition of a central memory phenotype by antigen-activated CD8 T cells cultured in IL-15.

## Discussion

We show that the differentiation of antigen-activated CD8 T cells into memory phenotypes through IL-15 or IL-2 signaling is subject to regulation by a limited number of microRNAs (Fig. 4,A). This is surprising in light of (a) the complex and still little understood transcriptional programs that drive fate determination in memory T cells, and (b) the fact that IL-15 and IL-2 impart signals to antigen-activated T cells through the common IL-2/IL-15 receptor βγ chain.

A first important consideration from our analysis is that the separate fate of central memory and effector memory T cells dictated by cytokine signaling is under coordinated regulation by a discrete number of microRNAs. Since IL-15 and IL-2 signal through a common receptor, it would appear that intensity rather than quality of signal determines regulation by miR-150 and miR-155 in activated CD8 T cells destined to acquire central memory T cell characteristics. Complex transcriptional events must take place to orchestrate repression of proliferation in CD8 T cells committed to central memory fate determination through IL-15. By comparing F5 and OT-I T cells it appears that the central memory phenotype is acquired within 48 hrs from discontinuing contact with antigen, and it stabilizes over the following days (Figure S1). They also show that different transgenic T cells undergo fate determination at a different pace, suggesting that in a polyclonal population, lineage differentiation of memory CD8 T cells may be a stochastic event.

A second important consideration underscored by this study is a pattern of reciprocal regulation between miR-150 and miR-155 in memory CD8 T cell fate determination. A reciprocal miR-150^low^/miR-155^high^ pattern of regulation was previously reported in B cell chronic lymphocytic leukemia [32]. Both miRNAs have also been linked to regulation of the immune response as demonstrated by genetic deletion and transgenic approaches. Of interest, miR-150 is suppressed upon T cell activation [33], underscoring the importance of a differential increase in miR-150 along the trajectory of central memory T cells (Fig. 4). In converse, miR-155, which is elevated in activated T cells, decreases in central memory T cells. Taken together, these lines of evidence suggest that a reciprocal miR-150^high^/miR-155^low^ regulation guides T cells along the central memory trajectory. We see this as the effort of emerging memory T cells to distance themselves from the activation state, rolling into a maintenance state (self-renewal). This is consistent with the fact that microRNAs of the let-7 family are down-regulated during IL-15-driven central memory fate determination (Fig. 2). Since a decrease in expression of let-7, a family of microRNAs tightly regulated during embryonic stem cell differentiation [34], enables self-renewal in embryonic stem cells [35], it appears as if self-renewal in incipient central memory T cells may result from a concomitant suppression of the let-7 family of microRNAs.

A third and novel finding of this study is the identification of KChIP.1 as a gene specifically transcribed during the differentiation of activated CD8 T cells into central memory T cells (Fig. 5, A and B). KChIP.1 belong to a family of Ca^2+^ -binding proteins that associate with the intra-cellular N-terminal domain of voltage-gated K^+^ channels [25]. Mouse T lymphocytes express two types of K^+^ channels originally termed “n” (normal) and “l” (large) [36]. The latter is upregulated upon activation by mitogens. By analogy with human K^+^ channels, the activatable K^+^ channel corresponds to the calcium-activated K^+^ channel (IKCa1) whose expression is greatly increased upon protracted activation through the TCR [37]. In contrast, the number of voltage-gated K^+^ channels (e.g., Kv1.3) is upregulated to 1,500-2,000 channels/cell [38]. In humans Kv1.3 is expressed in effector memory CD4 T cells, whereas central memory CD4 T cells express IKCa1 [39]. Thus, KChIP.1 transcriptional activation appears to hallmark a change in K^+^ channel expression in developing central memory T cells. KChIPs are also related to the downstream regulatory element antagonist modulator (DREAM), also a Ca^2+^-dependent transcriptional repressor expressed in the brain, thyroid gland and thymus [40]. Transgenic mice expressing a cross-dominant active DREAM and KChIPs show reduced T cell proliferation and decreased IL-2 production after polyclonal activation or TCR activation by antigen [26]. Thus, an elevated KChIP.1 in central but not effector memory T cells could function as Ca^2+−^-dependent repressor of proliferation favoring self-renewal/homeostatic proliferation in cells that need to be stored in secondary lymphoid organs until re-encounter with antigen. This is consistent with the fact that fate determination is subject to complex regulatory events involving miR-150 which has an early, direct effect on KChIP.1 expression (Figure S3).

The emergence of central memory T cells after antigen activation followed by IL-15 signaling suggests that the existence of a highly responsive and regulatable transcriptional machinery. IL-15 provides activated T cells with appropriate survival and proliferation signals [41], [42] and also prevents apoptosis [43]. Of note, IL-15 added 48 hrs after activation by antigen has important effects such as reduction of cell size, decrease in protein content and synthesis, and less efficient maintenance of IL-2R levels when compared with IL-2-directed memory T cell generation [44]. Since IL-15 (not IL-2) is associated with the transcriptional activation of KChIP.1 in antigen-activated T cells, and the elevated expression of KChIP.1 has been reported to restrain proliferation and IL-2 production by in activated T cells [26], it appears as if by lowering the metabolic demand of the cell and by restraining IL-2 mediated proliferation, the coordinated action of IL-15 and KChIP.1 prepares the cell for survival and self-renewal.

The present findings bear implications for the lineage differentiation problem. We show that activation of naïve T cells without effector stage differentiation suffices to set in motion the transcriptional program leading to central memory fate determination, and involves a discrete number of microRNAs. Arguably, regulation at this early stage is effective since the T cell (a) has yet to switch on the transcriptional program of the effector stage, and (b) needs not to deal with the pro-apoptotic consequences of the contraction phase. This view is at variance with the conclusion of T cell marking experiments [13], [14], [15] where memory T cells are progeny of effector cells which acquire self-renewal properties [45]. We posit instead that activated T cells committed to a central memory fate (T_CM_ precursors) activate a program that enables the acquisition of central memory phenotype through the balancing effects of miR-150/miR-155, and self-renewal characteristics through a decrease expression of let-7 microRNAs, a shared trait between embryonic stem cells and central memory T cells. These effects are synergistic and not absolute, and depend on a gradient effect similar to that observed for the transcription factor T-bet whose restrained expression during low inflammatory conditions is important to establish a memory CD8 T cell population [46]. Of note, miR-146, which is involved in Th1/Th2 differentiation and is a putative negative regulator of inflammatory conditions [47], was found to be down-regulated late in effector but not central CD8 T cells. Thus, our data suggest that cell fate determination in memory CD8 T cells operates according to an expression gradient of specific microRNAs. This does not exclude a synergy between microRNA regulation and regulation by other transcription factors [48] found to influence memory CD8 T cell emergence based on their relative expression [49].

In conclusion, here we show a new aspect in the biology of lineage differentiation of central memory CD8 T cells, regulation by micro-RNAs in cell fate determination at the pre-effector stage. The present findings are conceptually relevant for the design of vaccination strategies to more effectively select for the induction of protective central memory CD8 T cells [10].

## Materials and Methods

### Mice, synthetic peptides and immunological reagents

Eight wk old C57BL/6 mice were purchased from the Jackson Laboratories. F5 RAG^−/−^ TCR were obtained from the National Institute of Health (Bethesda, MD) through the courtesy of Dr. Jonathan Yewdell. OT-I C57BL/6 (Thy1.2^+^) were maintained in the animal facility of the University of California, San Diego. All animals were handled in strict accordance with good animal practice as defined by the relevant national and/or local animal welfare bodies, and all animal work was performed based on approved protocol by the Institutional Animal Subject Committee (UCSD No. S00023). Influenza virus NP_ 366–374_ peptide (ASNENMDAM) and Ova_257–264_ peptide (SIINFEKL) were synthesized at the Peptide Synthesis Core Facility of Ohio State University. rIL-15 was purchased from R&D and rIL-2 from eBioscience (San Diego, CA). Mitomycin-C and LPS were purchased from Sigma.

### Cell culture and transfection

#### LPS blast preparation

C57BL/6 spleens were harvested and 4×10^7^ cells/flasks cultured with LPS at a final concentration of 25 µg/ml plus dextran-sulfate at 0.7 mg/ml for 3 days. *Peptide pulsing of LPS blasts.* LPS blasts were harvested, treated with mitomycin- C (50 µg/ml) for 20 mins at 37°C, and pulse-labeled with OVA_257–264_ peptide or NP_ 366–374_ peptide at a final concentration of 10 µg/ml for 1 h at 37°C in a water bath.

#### Co-culture of naïve TCR transgenic cells with peptide-pulsed LPS

OT-I or F5 transgenic T cells were harvested and cultured with peptide-pulsed LPS blasts at 1×10^6^ TCR transgenic T cells/8×10^6^ LPS blasts per well (24-well plate) in 2 ml final volume of complete RPMI (10% FBS, RPMI-1640, penicillin/streptomycin, L-glutamine, NaPyr, NEAA, 2-ME, gentamicin and HEPES). On day 2 after co-culturing, the cells were harvested, dead cells removed using Lympholyte-M, and the remaining cells resuspended at 1 ml/well of complete RPMI. rIL-15 or rIL-2 were added at the final concentration of 40 ng/ml. Cells were incubated at 37°C in 5% CO_2_ for 6 days. Fresh rIL-15/rIL-2 was added to cultures every 2 days at a final concentration of 20 ng/ml. On day 8, cells were harvested and aliquots prepared for Flow cytometry analysis and miRNA extraction.

### Transfection

After Lympholyte M, cells were resuspended in 428 µl/sample of RPMI-1640 containing HEPES, L-glutamine, penicillin and streptomycin and plated in a 24-well plate. Lipofectamine™ transfection was performed according to the manufacturer's instructions. Briefly, lipoplexes were prepared by combining 4 µl Plus ™ reagents, 0.4 µg of plasmid DNA, 15 µl of (anti/pre)miRs (150 nM final concentration) and 25 µl of Optimem media per sample and incubated for 15 min at room temperature. Anti-miR-150 and pre-miR-150 were purchased from Applied Biosystem (Life Technologies, Carlsbad, CA). Per manufacrturer's specifications anti-miR™ microRNA inhibitors are chemically modified, single stranded nucleic acids designed to specifically bind to and inhibit endogenous microRNA molecules, whereas pre-miR™ microRNA precursor molecules are small, chemically modified double-stranded RNA molecules designed to mimic endogenous mature microRNAs. This solution was mixed with 2 µl of Lipofectamine in 25 µl of Optimem media. Lipoplexes were then combined and further incubated for 15 mins at room temperature. Following incubation, the DNA-miR-Plus™- Lipofectamine™ complexes were added to each well to a final volume of 500 µl. After 3 h incubation, media was removed and replaced with 2 mL of cRPMI and cytokine solution. Cytokines were replenished every two days as previously stated and the cells harvested at each point for analysis.

### Flow Cytometry

Culture CD8 T cells were stained using the following fluorochrome-conjugated anti-mouse antibodies: CD8 (Ly-2), CD44 (IM7), CD62L (MEL-14), CD43 (IB11), CD45R (B220), CD25 (PC61) and CCR7 (4B12). All antibodies were purchased from eBioscience. Viable T cells were analyzed by gating on the 7AAD negative (Calbiochem) and FITC-CD8^+^ population. Within the CD8+ viable cells, the central memory T cell population was identified as APC-CD62L^hi^/PE-CD44^hi^ and the effector memory as APC-CD62L^low^/PE-CD44^hi^. Flow cytometry was performed on a FACSCalibur (BD Biosciences, San Jose, CA). Data were acquired using BD CellQuest Pro and analyzed using FlowJo software (Tree Star, Ashland, OR).

### MicroRNA array

MicroRNA array analysis was performed as follows. Briefly, 5 µg of total RNA were used for hybridization of miR-NA microarray chips. These chips contain gene-specific oligonucleotide probes, spotted by contacting technologies and covalently attached to a polymeric matrix. The microarrays were hybridized in 6× SSPE (0.9 M NaCl/60 mM NaH2PO4 ·H2O/8 mM EDTA, pH 7.4)/30% formamide at 25°C for 18 hr, washed in 0.75× TNT (Tris·HCl/NaCl/Tween 20) at 37°C for 40 min, and processed by using a method of detection of the biotin-containing transcripts by streptavidin-Alexa647 conjugate. Processed slides were scanned using a microarray scanner (Axon), with the laser set to 635 nm, at fixed PMT setting, and a scan resolution of 10 mm. Microarray images were analyzed by using GenePix Pro and post-processing was performed essentially as described earlier [50]. Briefly, average values of the replicate spots of each miR-NA were background-subtracted and subject to further analysis. MicroRNAs were retained when present in at least 20% of samples and when at least 20% of the miR-NA had fold change of more than 1.5 from the gene median. Absent calls were thresholded prior to normalization and statistical analysis. Normalization was performed by using the quantiles method. MicroRNA nomenclature was according to the microRNA database at Sanger Center [51]. We identified genes that were differentially expressed among the two IL-15 and IL2 classes using paired t-test. Data have been deposited in a MIAME compliant database. The accession No. will be provided as it becomes available.

### Quantitative PCR (qPCR)

Total RNA was extracted from 4×10^5^ T cells with Applied Biosystems (ABI) miR-VANA miR-NA Isolation Kit (#AM1560) using the total RNA isolation procedure. Reverse transcription was performed from 250 ng of total RNA using High Capacity cDNA Reverse Transcription kit (ABI, 4368814) and 1 cycle at 25°C 10 min, 37°C 120 min, 85°C 5 s and 4°C hold. Gene specific primers mmu-miR-150#, mmu-miR-155 and snoRNA202 (ABI) were utilized to generate cDNA for miR-150, miR-155 and snoRNA202 (endogenous control). MicroRNAs were analyzed by duplicate qPCR following ABI protocol for master mix. 5 uL Taqman master mix, 0.5 uL probe, 1 uL cDNA and 3.5 uL water for a total reaction volume of 10 uL. Universal cycling conditions for qPCR were utilized consisting of 1 cycle of 50°C 2 min, 95°C 10 min followed by 50 cycles at 95°C 15 s, 60°C 60 s. Taqman inventoried and validated probe and primer set for mmu-miR-150, mmu-miR-155 and snoRNA202 were utilized for qPCR.

## Supporting Information

Figure S1 Kinetics of phenotypic changes in central memory development originating from antigen activated OT-I and F5 CD8 T cells. Spleens from naïve OT-I and F5 cells transgenic mice were harvested and cultured with LPS blasts pulsed with peptide (Ova257–264 for OT-I or NP366–374 for F5). After 48 hrs, LPS blasts were removed and activated cells were re-cultured with either IL-15 (40 ng/mL) or IL-2 (40 ng/mL). Every 48 hrs the media was replenished with fresh cytokines at 20 ng/mL. Expression of CD44 (IM7) and CD62L (MEL-14) were detected using anti-mouse CD44-PE and rat anti-mouse CD62L-APC. Cells were stained with 7-ADD (7-ADD PerCP) to identify dead cells, then gated on CD8 T cells. Each histogram represents the analysis of 10,000 7-ADD-negative and CD8 T cells. Numbers on the histograms represent percentage of cells in the respective quadrants.
(0.29 MB TIF)

Figure S2 Time course of microRNA variations in fate determination of memory CD8 T cells. (A) MicroRNA analysis of memory CD8 T cells cultured in IL-15 after antigen priming. Total RNA was extracted from cells at different time points after the initiation (day2) of IL-15 treatment. Samples were run in triplicate. Six independent experiments were performed, three with F5 and three with OTI CD8 T cells. For each time point, day 4 (D4), day 6 (D6), day 8 (D8) of treatment Cluster and TreeView were used to plot the log2 of fold changes relative to the control day 2 (D2). Red square means higher miRNA expression upon IL-15 and green squares mean lower miRNA expression upon IL-15 treatment. Black squares mean no change in expression. (B) MicroRNA analysis of memory CD8 T cells cultured in IL-2 after antigen priming. Total RNA was extracted from cells at different time points for IL-2 treatments. Six independent experiments were performed, three with F5 and three with OTI CD8 T cells. For each time point, day 4 day 4 (D4), day 6 (D6), day 8 (D8) of treatment Cluster and TreeView were used to plot the l log2 of fold changes relative to the control day 2 (D2). Red square means higher miRNA expression upon IL-2 and green squares mean lower miRNA expression upon IL-2 treatment. Black squares mean no change in expression. Samples were run in triplicate.
(0.26 MB TIF)

Figure S3 Anti-miR-150 accelerates the transcriptional activation of ChIP1 in celntral memory cells. Spleen cells from naïve OT-I transgenic mice were cultured with LPS blasts pulsed with peptide (Ova257–264). After 48 hrs, LPS blasts were removed and activated cells were were transfected with AM150 (150 nM) and cultured as indicated in Methods and further cultured with IL-15 (40 ng/mL). Two and six day later cells were harvested, the RNA extracted and KChIP.1 amplified using custom-made probes and primer set. Tests were done in triplicate.
(0.12 MB TIF)
