# Supplementary figures and images for: Selected MicroRNAs Define Cell Fate Determination of Murine Central Memory CD8 T Cells
Source: PLoS One. 2010 Jun 22;5(6):e11243. doi: 10.1371/journal.pone.0011243 (PMC2889817; doi:10.1371/journal.pone.0011243)

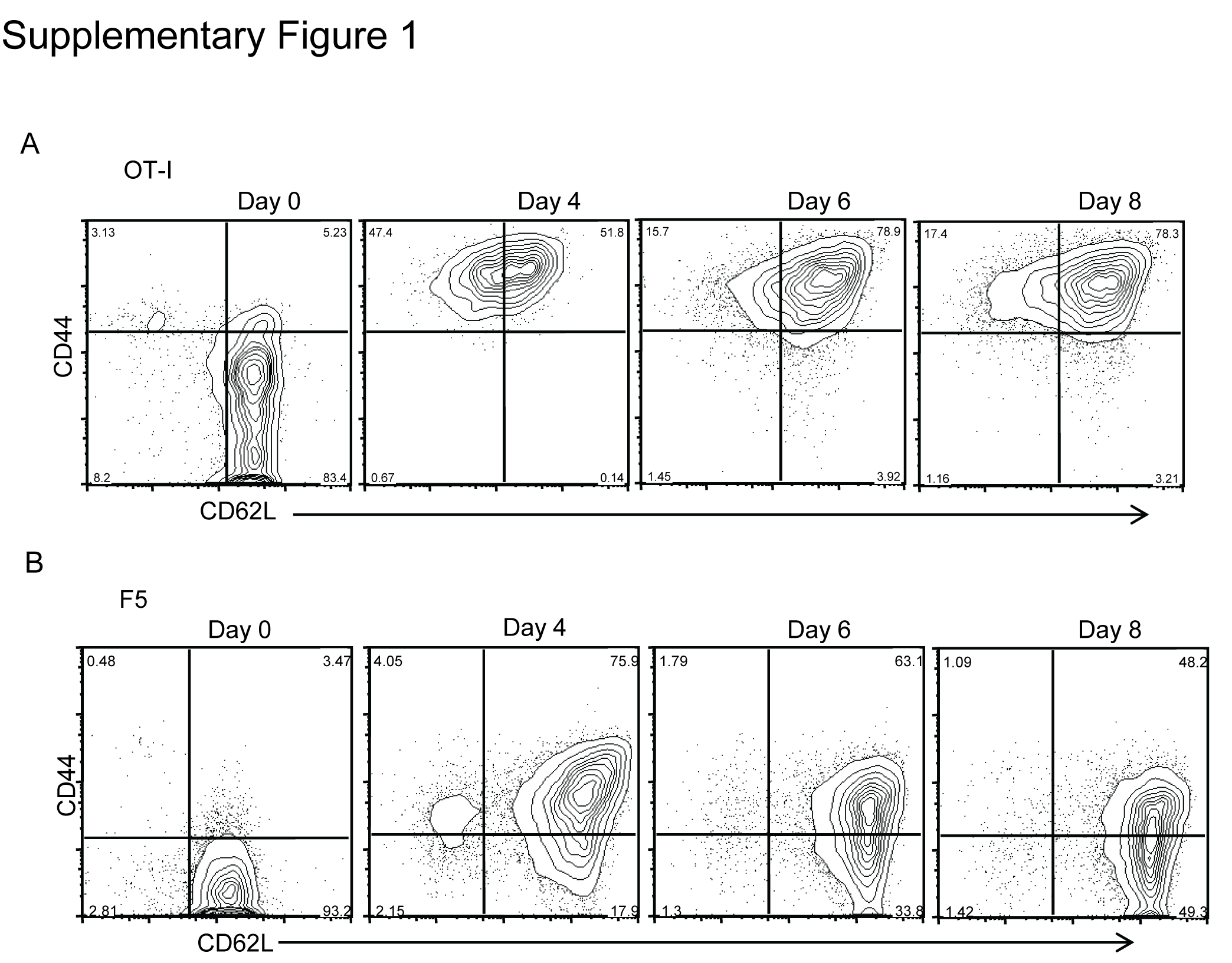

Supplement: Figure S1 — Kinetics of phenotypic changes in central memory development originating from antigen activated OT-I and F5 CD8 T cells. Spleens from naïve OT-I and F5 cells transgenic mice were harvested and cultured with LPS blasts pulsed with peptide (Ova257–264 for OT-I or NP366–374 for F5). After 48 hrs, LPS blasts were removed and activated cells were re-cultured with either IL-15 (40 ng/mL) or IL-2 (40 ng/mL). Every 48 hrs the media was replenished with fresh cytokines at 20 ng/mL. Expression of CD44 (IM7) and CD62L (MEL-14) were detected using anti-mouse CD44-PE and rat anti-mouse CD62L-APC. Cells were stained with 7-ADD (7-ADD PerCP) to identify dead cells, then gated on CD8 T cells. Each histogram represents the analysis of 10,000 7-ADD-negative and CD8 T cells. Numbers on the histograms represent percentage of cells in the respective quadrants. (0.29 MB TIF) [file pone.0011243.s001.tif]

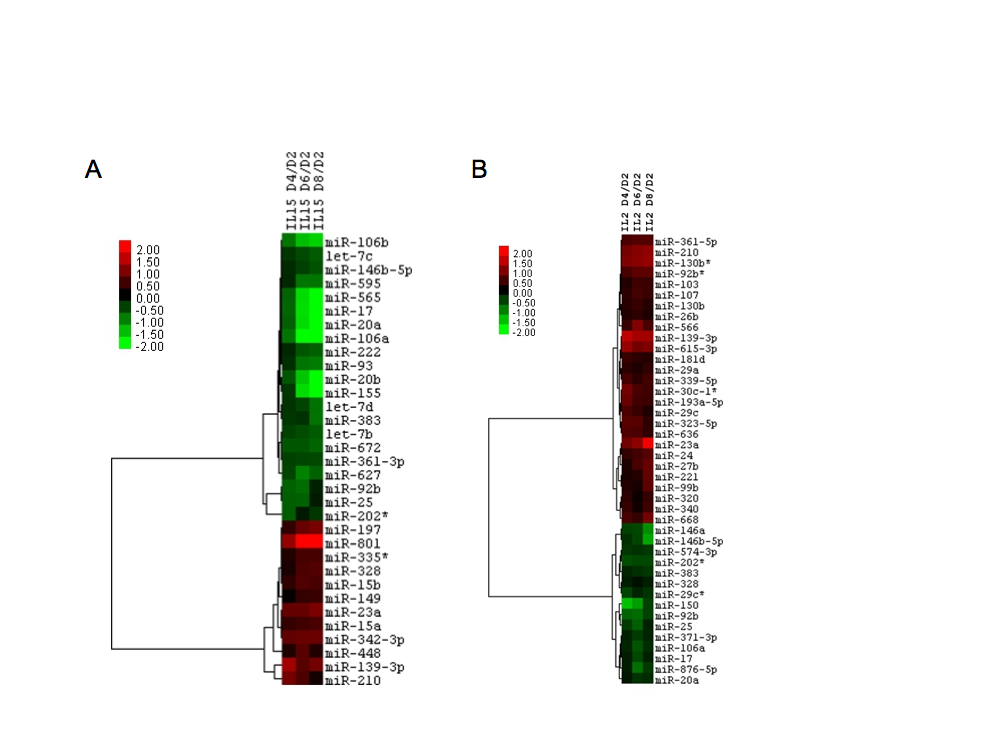

Supplement: Figure S2 — Time course of microRNA variations in fate determination of memory CD8 T cells. (A) MicroRNA analysis of memory CD8 T cells cultured in IL-15 after antigen priming. Total RNA was extracted from cells at different time points after the initiation (day2) of IL-15 treatment. Samples were run in triplicate. Six independent experiments were performed, three with F5 and three with OTI CD8 T cells. For each time point, day 4 (D4), day 6 (D6), day 8 (D8) of treatment Cluster and TreeView were used to plot the log2 of fold changes relative to the control day 2 (D2). Red square means higher miRNA expression upon IL-15 and green squares mean lower miRNA expression upon IL-15 treatment. Black squares mean no change in expression. (B) MicroRNA analysis of memory CD8 T cells cultured in IL-2 after antigen priming. Total RNA was extracted from cells at different time points for IL-2 treatments. Six independent experiments were performed, three with F5 and three with OTI CD8 T cells. For each time point, day 4 day 4 (D4), day 6 (D6), day 8 (D8) of treatment Cluster and TreeView were used to plot the l log2 of fold changes relative to the control day 2 (D2). Red square means higher miRNA expression upon IL-2 and green squares mean lower miRNA expression upon IL-2 treatment. Black squares mean no change in expression. Samples were run in triplicate. (0.26 MB TIF) [file pone.0011243.s002.tif]

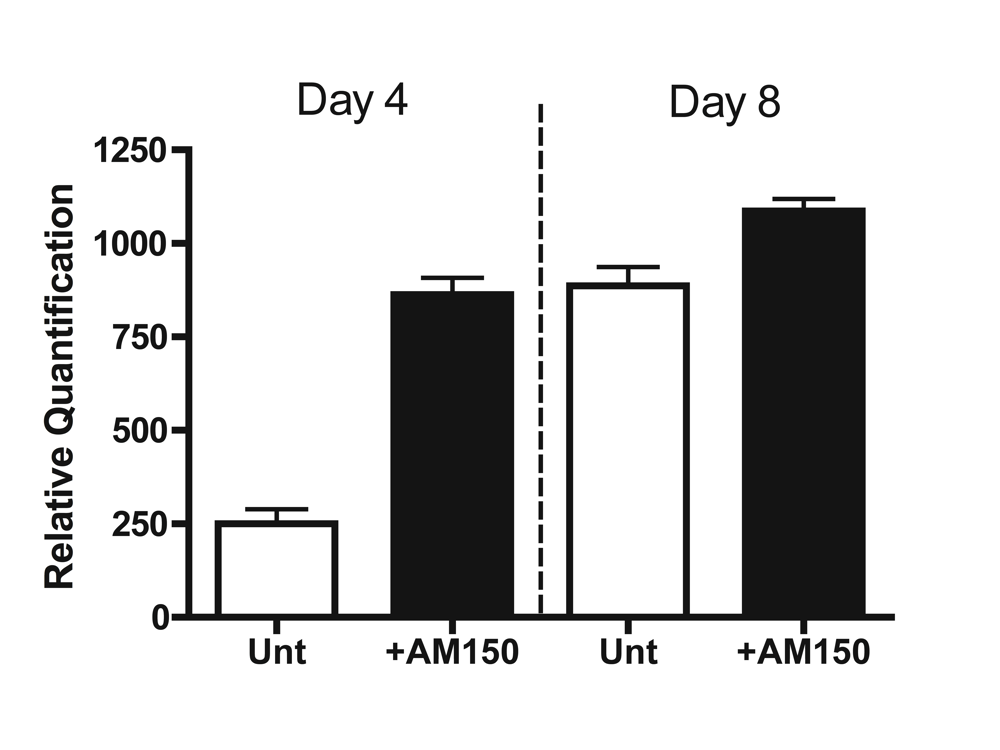

Supplement: Figure S3 — Anti-miR-150 accelerates the transcriptional activation of ChIP1 in celntral memory cells. Spleen cells from naïve OT-I transgenic mice were cultured with LPS blasts pulsed with peptide (Ova257–264). After 48 hrs, LPS blasts were removed and activated cells were were transfected with AM150 (150 nM) and cultured as indicated in Methods and further cultured with IL-15 (40 ng/mL). Two and six day later cells were harvested, the RNA extracted and KChIP.1 amplified using custom-made probes and primer set. Tests were done in triplicate. (0.12 MB TIF) [file pone.0011243.s003.tif]
